# Autism traits outweigh alexithymia traits in the explanation of mentalising performance in adults with autism but not in adults with rejected autism diagnosis

**DOI:** 10.1186/s13229-022-00510-9

**Published:** 2022-07-08

**Authors:** Christine M. Falter-Wagner, Carola Bloch, Lana Burghof, Fritz-Georg Lehnhardt, Kai Vogeley

**Affiliations:** 1 Department of Psychiatry and Psychotherapy, Medical Faculty, LMU Munich, Nussbaumstr. 7, 80336 Munich, Germany; 2 Department of Psychiatry and Psychotherapy, Faculty of Medicine and University Hospital Cologne, University of Cologne, Cologne, Germany; 3 Cognitive Neuroscience, Institute of Neuroscience and Medicine (INM-3), Forschungszentrum Jülich, Jülich, Germany

**Keywords:** Autism, Alexithymia, Mentalising, Dominance analysis

## Abstract

**Background:**

Pronounced alexithymia traits have been found in autism spectrum disorder (ASD) and recent research has been carving out the impact alexithymia traits might have on mentalising deficits associated with ASD.

**Method:**

In this cross-sectional study, a large representative referral population for diagnostic examination for possible ASD (*n* = 400) was screened for clinical alexithymia with a German version of the Reading the Mind in the Eyes test (RME). In contrast to previous attempts to carve out the impact of alexithymia traits on mentalising deficits though, we employed dominance analysis to account for the correlation between predictors. The *relative* relationship between alexithymia traits and autism traits with RME performance was investigated in the group of individuals with confirmed ASD diagnosis (*N* = 281) and compared to the clinical referral sample in which ASD was ruled out (*N* = 119).

**Results:**

Dominance analysis revealed autism traits to be the strongest predictor for reduced mentalising skills in the ASD sample, whereas alexithymia contributed significantly less. In the sample of individuals with ruled out diagnosis, autism traits were the strongest predictor, but alexithymia traits were in sum equally associated to mentalising, with the *External-Oriented Thinking* subscale as an important predictor of this association.

**Limitations:**

It needs to be considered that the cross-sectional study design does not allow for causal inference. Furthermore, mentalising is a highly facetted capacity and measurements need to reduce this complexity into simple quantities which limits the generalizability of results.

**Discussion:**

While alexithymia traits should be considered for their mental health importance, they do not dominate the explanation of reduced mentalising skills in individuals with ASD, but they might do to a larger degree in individuals with ruled out ASD.

**Supplementary Information:**

The online version contains supplementary material available at 10.1186/s13229-022-00510-9.

## Background

Autism spectrum disorder (ASD) is characterised by pervasive difficulties in communication and social interaction, as well as restricted and repetitive behaviour [1]. During the past decade, a psychopathological construct that oftentimes co-occurs with ASD caught scientific attention, referred to as *alexithymia*, which was first described by Sifneos [2] and shows a general population prevalence of 10–20% [3–7]. Within samples of individuals with ASD, the prevalence has been meta-analytically shown to be significantly increased to 50% [8]. Measures of alexithymia include three components: *Difficulties Identifying Feelings* (DIF), *Difficulties Describing Feelings* (DDF), and *Externally-Oriented Thinking* (EOT) [9, 10]. Alexithymia is relevant for its high prevalence in ASD and its role in comorbidities such as depression [11–15], anxiety [16], as well as autism symptomatology related to emotional processing [4, 17–19].

Yet, an important question beyond the clinical relevance of alexithymia traits for comorbidities is the relationship between alexithymia and mentalising. A recent review of the relationship between alexithymia and mentalising reported mixed results overall, due to sampling and differences in the measurement of mentalising, which may have affected the extent to which mentalising tests co-measured socio-affective skills [20]. In this context, one line of research supposes that socio-affective deficits observed in ASD might be due to co-occurring alexithymia, referred to as the so-called ‘alexithymia hypothesis’ [4]. Indeed, two recent studies suggested an association of alexithymia and attenuated performance on a popular mentalising task, the *Reading the Mind in the Eyes* (RME) test [21] in adults with ASD and an alexithymia-matched comparison group. Performance depended on the degree of alexithymia, but not on diagnostic group affiliation, further, alexithymia scores outweighed autism traits in the prediction of RME scores employing hierarchical regression [22]. The same dataset showed a negative correlation between alexithymia and RME scores without group interaction [23], which was not the case for a different measure of mentalising (i.e., emotional scale of the Movie for Assessment of Social Cognition [24]). The authors therefore suggested that lower RME performance may not be indicative for ASD diagnosis, but rather be explained by co-occurring alexithymia in ASD samples. So besides the fact that the RME has been frequently used as an index of mentalising abilities in autism research [25], RME performance may in part display emotion recognition abilities, as postulated by Oakley et al. [22] and Pisani et al. introduced a taxonomy of mentalising measures and suggested to refer to RME as a measure of mentalising abilities with emotional demand that may be substantially influenced by emotion recognition abilities [20. However, the authors further report that studies with clinical populations did not always report negative associations of alexithymia with RME, indicating possibly distinct strategies that impacted the extent to which emotion recognition abilities affected RME performance and a need for further investigations in large datasets.

Apart from the important contribution of previous studies for our understanding of the relationship of alexithymia with mentalising, the relative effects of autism traits and alexithymia traits are not necessarily assessable by regression weights due to the strong covariation of alexithymia and autism traits in individuals with ASD [26–29]. Thus, an adequate analysis of continuous effects of alexithymia and autism traits on mentalising that can account for the strong covariation is required. Moreover, Demers and Koven [30] showed that only EOT was negatively associated with RME scores in a control sample, which was also found in Lyvers et al. [31]. This indicates that splitting alexithymia into subfactors can be informative when investigating differential effects of alexithymia on mentalising performance, as a characteristic autism trait. In light of their extensive systematic review in this field, Pisani et al. further highlight the importance to investigate the relationship of alexithymia and mentalising while controlling for influencing factors such as intelligence, executive functions, and verbal abilities [20].

Consequently, the first aim of the current study was to investigate whether either autism traits or alexithymia traits might predict mentalising skills more strongly by means of dominance analysis while taking important covariates into account.

Moreover, if the previously reported association between alexithymia and mentalising [22, 23] is confirmed in the current representative referral population of individuals who presented with social interaction difficulties at an autism outpatient clinic without final ASD diagnosis, this would imply that individuals without ASD, but with alexithymia, may share some of the autistic clinical picture. This would potentially add to the challenge of differential diagnostics of ASD, which is particularly difficult in adulthood [32]. Thus, the second aim of the current study was to investigate whether the pattern of association between alexithymia traits and autism traits with mentalising skills might differ between individuals with confirmed ASD and a clinical comparison sample with ruled out ASD diagnosis, both drawn from a representative referral population for ASD diagnostic clarification.

## Method

### Participants

Data from the referral population of the outpatient clinic for autism in adulthood at the University Hospital Cologne were sampled post hoc from referrals in the period 2006–2019 (see Table 1). Ethical approval was granted by the ethics committee of the medical faculty, University of Cologne (20-1432). Patients were referred to the outpatient clinic by medical consultants for the purpose of diagnostic clarification due to self-reported social-emotional symptoms. Diagnostic procedures were in accordance to German guidelines on ASD [33], including neuropsychological testing and consensus diagnostics based on the independent assessment by a minimum of two experienced clinicians.

**Table 1:** Characteristics of participants with (ASD+) and without (ASD−) ASD

	**ASD+**			**ASD−**					
	*N*	**%**		*N*	*%*		*χ*^2^	*df*	*p*
Male	219	78		81	68				
Female	62	22		38	32		3.832	1	.050
	*M*	*SD*	**Range**	*M*	*SD*	**Range**	*t*(398)	*p*	*d*
Age	33.2	11.0	18–71	33.5	12.5	18–74	0.253	.801	.027
FIQ	104.3	16.6	71–152	100.7	14.7	73–146	− 2.055	.041	− .230
PIQ	99.7	16.3	62–148	98.0	14.1	57–136	− 1.040	.299	− .117
VIQ	107.0	16.4	64–144	101.8	15.2	69–139	− 2.942	.003	− .326

Number and percentage of males and females in groups with results of chi-square test for frequency distribution. Mean parameters (*M*) with standard deviations (*SD*) for Age, Full-Scale IQ (FIQ), Performance-based IQ (PIQ), and Verbal IQ (VIQ), and results of group comparisons with Student's *t*-tests with effect size Cohens *d*.

Individuals with confirmed ASD (ASD+; *N* = 281) received a diagnosis of F84.5 (*n* = 242; *Asperger Syndrome*), F84.1 (*n* = 18; *Atypical Autism*), or F84.0 (*n* = 21; *Childhood Autism*) according to ICD-10 [28]. This group was compared to individuals from the referral population for who any diagnosis of F84 was ruled out (ASD-; *N* = 119).

The standard clinical neuropsychological assessment included, amongst others, the *Autism Spectrum Quotient* (AQ) [34], the *20-Item Toronto Alexithymia Scale* (TAS-20) [10], and the *Wechsler Adult Intelligence Scale* WIE-III [35]. Inclusion criteria for sampling were cases with total IQ scores > 70 as well as < 4 missing items on the relevant questionnaires. For the AQ, one to three missing item were recorded for *n* = 26 in the ASD+ sample and *n* = 17 in the ASD− sample. For the TAS-20, one to three missing items were recorded for *n* = 11 in the ASD+ sample and *n* = 4 in the ASD− sample. Missing items were filled by item means. In addition, one case (ASD+) was an extreme outlier on the AQ score (< 3 *SD* from *M*) and was therefore not included in the analysis.^1^

### Neuropsychological assessment

The revised version of the RME test measures mentalising abilities in adults and was introduced as a tool for ASD screening [36]. A modified German version of the test was used in this study [24, 37–40], consisting of 24 pictures of the eye region with different affective and cognitive expressions. Participants were instructed to choose the most appropriate label for the mental state of the presented person out of four possible suggestions. Correct answers were summed to a total score with a maximum of 24.

The AQ is a 50-item self-rating questionnaire of autism traits [34]. Responses are given on four-point Likert-scales (1 = strongly *agree* to 4 = strongly *disagree*). Based on the ratings, each item was recoded to values 1 or 0 and summed up to a maximum score of 50 [34]. Higher values indicate more pronounced autism traits. AQ Scores > 32 depict pronounced autism traits [34]. In the ASD+ 83.63% and in the ASD− group, 78.15% of cases reported autism traits above this threshold. The AQ showed good discriminant validity and retest-reliability [41]. Cronbach’s $\alpha$ revealed good internal consistency ($\alpha$ = .86).

The TAS-20 is a self-rating questionnaire of alexithymia traits comprising three factors: *Difficulties Identifying Feelings* (DIF), *Difficulties Describing Feelings* (DDF), and *Externally-Oriented Thinking* (EOT) [10]. Responses are given on five-point Likert-scales, ranging from 5 = *strongly agree* to 1 = *strongly disagree*. Five inverse items need to be recoded. All items are then summed up to a maximum score of 100 (*max* DIF: 35, *max* DDF: 25, *max* EOT: 40). The recommended cut-off for clinically relevant alexithymia is 61 [42]. The factor structure was confirmed in different languages [43] and showed good internal validity in a large German sample [7]. Cronbach’s $\alpha$ showed good internal consistencies for DIF ($\alpha$ = .85) and DDF ($\alpha$ = .74) and acceptable internal consistency for EOT ($\alpha$ = .60). Most importantly in this research context is a recent study showing that despite their frequently observed covariation, autism and alexithymia traits measured with AQ and TAS can be considered as distinct constructs [44].

### Statistical procedures

Data preprocessing and analysis were performed using RStudio [45] with a similar analysis approach as in our previous study [11].

For bivariate relationships, Pearson zero-order correlations of measures of interest were calculated (Table 2). For multivariate analysis, regression models were calculated with control variables, autism traits (AQ), and alexithymia traits (DIF, DDF, EOT) as predictors of mentalising scores (RME), separately for the ASD+ and ASD− samples (Table 3). The model controlled for variables age, sex, PIQ, and VIQ (Table 1). The model fit was adequate (< 6% of standardized residuals > 2), independence of errors was confirmed (Durbin-Watson statistic ≈ 2) and there was no multicollinearity (variance inflation factor < 2 for all predictors in all models). Heteroscedasticity was implied by residual plots and a significant result in the Breusch Pagan Test for both models. Robust regressions with heteroscedasticity-consistent variance covariance matrix (HCCM) were conducted, using the *sandwich* and *lmtest* packages in R [46, 47]. HCCMs were retrieved by the *vcovHC()* function, using HC3 method [48, 49].

**Table 2:** Descriptive statistics and correlations for variables of interest

Variable	Sample	*M*	*SD*	*range*	RME	AQ	DIF	DDF	EOT
RME	ASD+	15.1	4.5	0–23	–				
	ASD−	15.8	4.2	2–23					
AQ	ASD+	38.7	6.1	20–49	− .31***	–			
	ASD−	37.6	5.8	21–47	− .32***				
TAS-20 (DIF)	ASD+	22.1	6.1	7–35	− .17**	.52***	–		
	ASD−	23.2	6.6	7–35	− .24**	.44***			
TAS-20 (DDF)	ASD+	19.1	3.8	5–25	− .09	.47***	.59***	–	
	ASD−	18.9	4.0	5–25	− .22*	.47***	.61***		
TAS-20 (EOT)	ASD+	23.4	5.2	9–39	− .08	.00	.08	.22***	
	ASD−	23.7	5.1	9–40	− .19*	.02	− .01	.17	
TAS-20	ASD+	64.6	22.1	26–91	− .16**	.46***	.80***	.78***	.59***
	ASD−	65.8	23.2	30–90	− .31**	.44***	.81***	.80***	.51***

RME = German 24-item Reading the Mind in the Eyes test; AQ = Autism Spectrum Quotient; TAS-20 = 20-Item Toronto Alexithymia Scale; TAS-20 (DIF) = TAS-20 subscale *Difficulties Identifying Feelings*; TAS-20 (DDF) = TAS-20 subscale *Difficulties Describing Feelings*; TAS-20 (EOT) = TAS-20 subscale *Externally-Oriented Thinking*

*p < .05; **p < .01; ***p < .001

**Table 3:** Models with RME as dependent variable and results of dominance analysis

*Predictors*	ASD+				ASD -			
	*B*	*SE*_*adj*_	*GDW*	*LL, UL*	*B*	*SE*_*adj*_	*GDW*	*LL, UL*
*Age*	.002	.026	.002	.001, .029	− .044	.039	.025	.002, .117
*Sex*	.996	.662	.013	.001, .063	1.95^*^	.863	.054	.004, .197
*PIQ*	.017	.018	.005	.001, .025	.005	.030	.003	.001, .044
*VIQ*	.029	.018	.008	.001, .024	.025	.036	.011	.002, .075
AQ	− .271^***^	.060	.088	.035, .149	− .149	.082	.059	.015, .152
*DIF*	− .049	.053	.014	.003, .045	− .055	.078	.021	.005, .090
*DDF*	.170*	.081	.007	.003, .026	− .010	.102	.015	.005, .063
*EOT*	− .057	.051	.004	.000, .029	− .151*	.066	.032	.006, .089
*R*^2^			.142				.220	

PIQ = Performance-based IQ; VIQ = Verbal IQ; AQ = Autism Spectrum Quotient; DIF = TAS-20 subscale *Difficulties Identifying Feelings*; DDF = TAS-20 subscale *Difficulties Describing Feelings*; EOT = TAS-20 subscale *Externally-Oriented Thinking*

Regression model estimates with results of dominance analysis in ASD+ (*N* = 281) and ASD− (*N* = 119) samples. Lower limits (*LL*) and upper limits (*UL*) of bootstrapped confidence intervals for General Dominance Weights (*GDW*)

Results of *t*-tests of coefficients (*B*) after adjustment of errors (*SE*_adj_) by robust regression are reported by asterisks

*p < .05; **p < .01; ***p < .001

Correlations of AQ, DIF, DDF, and EOT could have resulted in problems when interpreting their relative effects by means of beta coefficients, whereas beta coefficients only represent total effects,Ggeneral Dominance Weights (*GDW*) constitute an index of importance for each predictor that was suggested as a new comparison standard in multiple regression because it considers direct, total and partial effects [50]. *GDW*s and bootstrapped 95% confidence intervals (100 resamples) were calculated with *yhat* package in R [51]. We applied dominance analysis to calculate *GDW*s. Unique variance explained by each predictor was calculated by the squared semipartial correlation averaged across all models in all possible subsets that included that predictor [50]. The total determination factor *R*^2^ of the model represents the sum of GDWs [28].

Additional tests of statistical significance at 0.05 level in form of deviance tests of nested multivariate models were conducted. Nullmodels that included covariates only were first compared to models including AQ (first comparison), and this model was compared to an additional model containing TAS full scores (second comparison). To account for the effect by the order of intake of predictor, in a third step, nullmodels were compared to models including TAS (first comparison) and these models were then compared to a model that additionally contained AQ (second comparison).

As an additional exploration that was suggested by a reviewer, dominance analysis was calculated with AQ subscales instead of AQ full-scale. Results of this analysis can be found in the Additional file 1.

## Results

The groups did not significantly differ with respect to our predictor variables (AQ, DIF, DDF, EOT; largest *t* = 1.698). Descriptive statistics and correlations of variables of interest are to be found in Table 2. DIF was significantly correlated with AQ in the ASD+ sample (*r* = .52, *p* < .001, 95% *CI* [.43, .60]) and in the ASD− sample (*r* = .44, *p* < .001, 95% *CI* [.28, .57]). The same was found for DDF, which significantly correlated with AQ in the ASD+ sample (*r* = .47, *p* < .001, 95% *CI* [.38, .56]) and in the ASD− sample (*r* = .47, *p* < .001, 95% *CI* [.32, .60]).

In the ASD+ sample, dominance analysis showed that the greatest proportion of variance in RME scores was explained by AQ (*GDW* = .088, CI [.035, .149]), followed by DIF, that had a noticeably smaller effect size (*GDW* = .014, CI [.003, .045]). DDF was ranked third (*GDW* = .007, CI [.003, .026]) and EOT fourth (*GDW* = .004, CI [.000, .029]) with respect to their explanatory power (Table 3).

Likewise, model estimates in the ASD− sample revealed AQ as the strongest predictor of variance in RME scores (*GDW* = .059, CI [.015, .152]), but in contrast to the ASD+ sample, EOT had the second largest weight (*GDW* = .032, CI [.006, .089]). DIF was ranked third (*GDW* = .021, CI [.005, .090]) and DDF weakest predictor (*GDW* = .015, CI [.005, .063]).

Deviance tests in the ASD+ sample showed that the inclusion of AQ significantly improved model fit (*F*(1) = 3.05, *p* < .001) and the additional inclusion of TAS did not further improve model fit beyond AQ (*F*(1) = 0.03, *p* = .853). With the order of inclusion changed, the inclusion of TAS did significantly improve model fit (*F*(1) = 5.62, *p* = .018), however, the additional inclusion of AQ still significantly improved the variance explanation (*F*(1) = 23.90, *p* < .001). In the ASD− sample, the first comparison showed that the AQ significantly improved the model fit (*F*(1) = 8.57, *p* = .004) and there was a marginal improvement by further inclusion of TAS (*F*(1) = 3.23, *p* = .075). Likewise, first including TAS scores significantly improved the variance explanation (*F*(1) = 8.81, *p* = .004) and the further inclusion of AQ marginally improved the model fit (*F*(1) = 3.00, *p* = .086).

Inclusion of the five subscales of the AQ instead of the total scale showed in the ASD+ group that compared to all subscales (i.e., AQ and TAS subscales), the AQ subscales *Attention to Detail*, *Communication*, and *Imagination* achieved the strongest weights, whereas in the ASD- group, it was the AQ subscale *Imagination* and the TAS subscale EOT, see Additional file 1.

## Discussion

While increasing evidence points to an important role of alexithymia traits for comorbidities in ASD [11, 16, 52], the focus in the current study was on whether alexithymia traits might also be responsible for mentalising as one characteristic autism trait. For this purpose, we targeted mentalising abilities and performed dominance analysis on the relative explanatory power of alexithymia traits (divided into three subdomains DIF, DDF, EOT) and autism traits in a large representative sample of adults referred to an autism outpatient clinic. We (i) replicated the result of a negative correlation between alexithymia and RME scores by Rødgaard et al. [23], yet furthermore found (ii) that autism traits outweighed alexithymia traits in explaining mentalising abilities in ASD deploying methodology that controls for the intersection of autism and alexithymia trait measures and (iii) that this was the case in both individuals with confirmed and ruled out ASD diagnosis (iv) with alexithymia traits showing a stronger association with mentalising in the diagnostic exclusion group, for which alexithymic traits may be considered, in the aggregate, to be a similarly good predictor for attenuated RME performance as autistic traits. The significance tests provided further evidence for a larger relevance of autism traits in the explanation of mentalising abilities in ASD, as the AQ significantly improved the models, irrespective of the order of inclusion, whereas TAS scores did not. However, in the ASD− sample, AQ and TAS scores showed similar significance patterns in the deviance tests.

Both groups showed similarly high autism and alexithymia traits in line with a recent meta-analysis [8]. The point prevalence of alexithymia was 66.19% in ASD+ and 68.91% in the ASD− group, compared to 10–20% for community samples [3–7].

Our results have direct relevance to the recently formulated ‘alexithymia hypothesis’ [4]. In their article, Bird and Cook argue that many tests of mentalising using emotional stimuli, such as the RME task [36], might yield attenuated performance in individuals with ASD due to confounding high traits of alexithymia in this population. While we agree that alexithymia should be carefully monitored in studies of ASD, our results indicate that, compared to autism traits, alexithymia contributes less to mentalising performance in ASD employing dominance analysis in a large representative sample of individuals with ASD. This is actually consistent with the alexithymia hypothesis, which refers exclusively to affective autism symptomatology, under the assumption that the RME actually measures mentalising in ASD. However, it contradicts with the assumption that RME measures predominantly emotion recognition in individuals with ASD that should have resulted in a stronger weighting of TAS.

Focusing on the different alexithymia components, DDF was a significant predictor of mentalising abilities in the ASD+ sample, but dominance analysis revealed a different weighting with a larger GDW for DIF and a clear dominance of AQ in comparison to both. Considering that dominance analysis controls for the correlations of predictors (which is not the case for regression coefficients), we built our inference on the predictor weighting by means of GDWs, aligning to previous approaches that deployed dominance analysis for predictor weighting [26, 53]. Thus, while alexithymia is to be taken into account due to its corroborated importance for anxiety [16] and depression in ASD [11], our results indicate that alexithymia’s influence does not explain mentalising when autism traits are additionally considered.

Our results are in accordance with previous studies showing only weak associations between alexithymia and mentalising skills [54, 55]. Furthermore, our results relate to previous findings of an association of alexithymia and reduced RME performance in ASD [22, 23] by showing in a large sample that autism traits outweighed alexithymia traits in the prediction of mentalising. As explained before, the large covariation of alexithymia and autism traits within ASD populations [26, 27] calls for a thoughtful choice of analysis methods. We chose dominance analysis as it is able to account for the *relative* effects of strongly covarying predictors. In particular, our finding of marked dominance of autism traits predicting mentalising skills was especially pronounced in the ASD+ sample.

Given that the large ASD and clinical comparison samples tested in this study did likewise not differ in the quantity of their autism traits and in the pattern of dominant prediction of RME performance by those autism traits indicates that differential diagnostics faces an equal challenge by both the high alexithymia traits and the high autism traits found in other clinical samples of individuals with socio-emotional difficulties but without ASD (see discussion in [56]).

However, regarding the results of deviance tests and dominance analysis in the ASD- sample, it should be highlighted that alexithymia and autism traits both contributed to RME performance which contrasts the pronounced dominance of autism traits that we found in the ASD sample. Moreover, the findings by Demers and Koven [30] and Lyvers et al. [31], showing that the subscale of the TAS-20 (*Externally-Oriented Thinking*, EOT) was negatively associated with RME scores in control samples, called for investigating the subscale dimensionality of the alexithymia scale and probably also the alexithymia construct per se. In the current study, we therefore considered the three TAS-20 subscales and found similar results. In relation to the other scales, EOT had the second largest effect on RME scores in the clinical comparison group tested in the current study. Demers and Koven [30] had argued for a conceptual differentiation of EOT from DIF and DDF, because of its stability over time [57] and its cognitive instead of affective characterization [30, 58]. In our ASD+ sample, however, it was DIF that was the second best predictor of mentalising skills after autism traits. While autism traits were the strongest predictor in both, some aspects of alexithymia contributed to mentalising performance in the RME task in both groups, and especially in the non-ASD sample. Arguably, the contribution of alexithymia aspects on mentalising abilities might be found even lower in tasks not drawing heavily on facial emotion recognition such as the RME task (as discussed by [22, 23]).

## Limitations

There were no control participants included in this study. Instead, individuals with confirmed ASD diagnosis were compared to a clinical group of individuals who were referred for a possible ASD diagnosis.

Furthermore, it has to be stated clearly that a cross-sectional study design as used in the current study does not allow for causal inference. Longitudinal studies are required to pin down causal directionality of effects and developmental relationships between alexithymia and autism. This implies though that alexithymia traits might be acquired along developmental pathways, another issue to be determined by future investigations.

Our aim was to investigate the relative explanatory contribution of alexithymia and autism traits to mentalising abilities, which was effectively measured by a modified proven German version [24, 37–40] of the RME test [21]. As the current RME version differed from the version in Oakley et al. [22], we note that a direct comparison of results is limited. Furthermore, Rødgaard et al. [23] reported no correlation of TAS scores with a different mentalising measure (i.e. emotional MASC scale [24]) that comprises video stimuli which might have facilitated mentalising due to additional contextual information. In this context, it is worth mentioning that mentalising is a high-level, multi-facetted capacity that incorporates various cognitive mechanisms and its neural correlates are distributed in multiple brain regions [59–63]. Assumingly, there is no measure that spans all facets of mentalising and that controls for all external factors on performance (e.g., verbal abilities, empathetic abilities). Thus, the generalisability and comparibilty of mentalising tests is always limited to the specific stimulus material and specificities of the test. However, it must be emphasized that there are innovative measurement tools for mentalisation that were recently developed and should be considered as an alternative to the RME in future studies [64–66].

Another limitation of the current study is the inclusion of a heterogeneous clinical controlcomparison sample due to naturalistic sampling from representative patient populations from outpatient clinic. The generalisability of the findings in the clinical comparison sample is therefore limited. Nonetheless, the heterogeneity of the clinical comparison sample does not impact the findings from dominance analysis within the ASD sample. And furthermore, the advantage of our clinical comparison sample is that it stems from a representative referral population at an outpatient clinic for autism and therefore is exactly the type of clinical group that needs to be differentiated in clinical reality. This type of comparison group shows various social interaction problems and is therefore phenomenologically very close to our ASD sample and hence our approach can be regarded as conservative.

## Conclusion

In conclusion, by deploying dominance analysis to infer the relative predictive power of alexithymia and autism traits in a large representative and homogeneous ASD sample and comparison to individuals with ruled out ASD from the referral population for autism diagnosis, the current study contributes to the differentiation between autism and alexithymia. By far the strongest relative predictor of mentalising skills were autism traits for individuals with ASD. To a far lesser extent, differential alexithymia subscales contributed to mentalising skills in ASD but in sum to a similar extent in a group of individuals with ruled out ASD diagnosis. Our results contribute to further differentiation of the alexithymia hypothesis of autism [4] in that alexithymia should be assessed for its high relevance for mental health issues in ASD [11, 16], but at the same time, our findings suggest autism traits to be the dominant predictor of mentalising as one characteristic autism trait.

## Supplementary Information

**Additional file 1**. Supplementary Analysis 1 and Supplementary Figure 1.

## Data Availability

The dataset analysed in this study is not publicly available because it contains clinical data from patient that are subject to privacy protections.

## Abbreviations

RME: Reading the Mind in the Eyes Test
AQ: Autism Spectrum Quotient
TAS-20: 20-Item Toronto Alexithymia Scale
DIF: Difficulties Identifying Feelings
DDF: Difficulties Describing Feelings
EOT: Externally-Oriented Thinking
VIQ: Verbal Intelligence Quotient
PIQ: Performance-based Intelligence Quotient

## Footnotes

1 Inclusion of this case in ASD + group did neither change significance of predictors in multiple regression nor did it change the pattern of results of dominance analysis.
