# Supplementary material for: Autism traits outweigh alexithymia traits in the explanation of mentalising performance in adults with autism but not in adults with rejected autism diagnosis
Source: Mol Autism. 2022 Jul 8;13:32. doi: 10.1186/s13229-022-00510-9 (PMC9264711; doi:10.1186/s13229-022-00510-9)
Supplement: Supplementary file 1 — Additional file 1. Supplementary Analysis 1 and Supplementary Figure 1. [file 13229_2022_510_MOESM1_ESM.docx]

**Supplementary Material**

**Supplementary Analysis 1**

The AQ was split into its five subscales (*social skill* (SoSk), *attention switching* (AttSw), *attention to detail* (AttDe), *communication* (Comm), *imagination* (Ima)) and the subscales were included in a linear model with the TAS subscales as predictors and age, gender, and IQ as covariates. We assumed that the subscale SoSk, with items such as ‘*I find it easy to work out what someone is thinking or feeling just by looking at their face*’ (reverse) or ‘*I find it difficult to work out people’s intentions*‘ would be especially predictive of decreased RME scores. Additionally, we assumed that the subscale AttDe with items like ‘*I usually concentrate more on the whole picture rather than the small details*’ (reverse) and ‘*I tend to notice details that others do not*’ may contribute to decreased RME performance due to altered mechanisms of visual perception of the eye pictures.

The results of the dominance analysis are depicted in Supplementary Figure 1. Results showed the following ranking of AQ and TAS subscales from strongest to weakest GDW in the ASD sample: AttDe (26.8% of R²), Comm (18.3% of R²), Ima (16.3% of R²), AttSw (6.5% of R²), DIF (5.9% of R²), SoSk (3.3% of R²) and EOT (3.3% of R²), and DDF (2.6% of R²). In the non-ASD sample results showed the following ranking of predictors from strongest to weakest GDW: Ima (17.7% of R²), EOT (12.1% of R²), Comm (8.6% of R²), DIF (8.2% of R²), SoSk (6.0% of R²), AttDe (5.2% of R²) and DDF (5.2% of R²), and AttSw (1.2% of R²).

Contrary to the a priori assumptions, SoSk was only sixth strongest predictor in the ASD sample and fifth strongest predictor in the non-ASD sample. As assumed, AttDe was strongest predictor for decreased RME scores in the ASD sample. Together with the AQ subscales Comm and Ima, AttDe thereby stands out from the other predictors by explaining 61.4% of R² in the ASD model.

The picture was different in the non-ASD sample, where the AQ subscale Ima was strongest predictor and TAS subscale EOT second strongest predictor, together accounting for 29.8% of explained variance by the model.

**Supplementary Figure 1**

General Dominance Weights (GDW) with bootstrapped confidence intervals as errorbars from dominance analysis with AQ subscales and TAS subscales in both samples


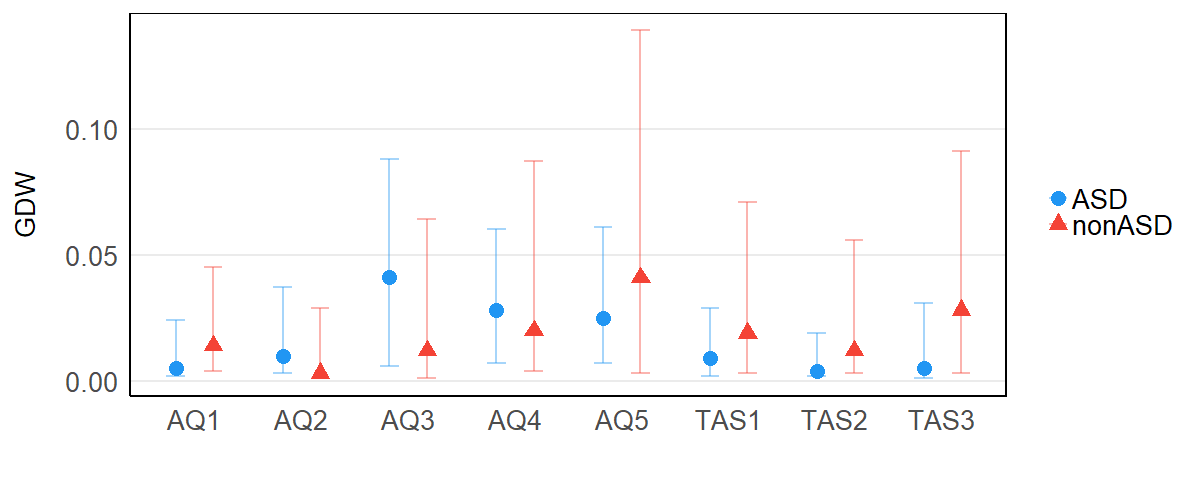


*Note.* AQ1 = *social skills*; AQ2 = *attention switching*; AQ3 = *attention to detail*, AQ 4 = c*ommunication*, AQ5 = *imagination*; TAS1 = *difficulties in identifying feelings*; TAS2 = *difficulties describing feelings*; TAS3 = *externally-oriented thinking*; GDW = *General Dominance Weights*. GDW were retrieved by dominance analysis, they sum up to total R² of models and thereby display relative proportions of variance explanation by predictors.
